# Supplementary material for: Self-cleavage of the GAIN domain of adhesion G protein-coupled receptors requires multiple domain-extrinsic factors
Source: Nat Commun. 2025 Oct 1;16:8736. doi: 10.1038/s41467-025-64589-3 (PMC12488864; doi:10.1038/s41467-025-64589-3)
Supplement: Supplementary file 1 — Supplementary Information [file 41467_2025_64589_MOESM1_ESM.docx]

**SUPPLEMENTARY INFORMATION**

TITLE

Self-cleavage of the GAIN domain of adhesion G protein-coupled receptors requires multiple domain-extrinsic factors

AUTHOR LIST

Yin Kwan Chung^1,2^, Christian H. Ihling^3,4^, Lina Zielke^1^, Signe Mathiasen^2^, Andrea Sinz^3,4^, Tobias Langenhan^1,5,6,*^

AFFILIATIONS

^1^ Rudolf Schönheimer Institute of Biochemistry, Division of General Biochemistry, Medical Faculty, Leipzig University, Johannisallee 30, 04103 Leipzig, Germany

^2^ Department of Biomedical Sciences, Faculty of Health and Medical Sciences, University of Copenhagen, Blegdamsvej 3B, 2200 Copenhagen, Denmark

^3^ Department of Pharmaceutical Chemistry and Bioanalytics, Martin-Luther-University Halle-Wittenberg, Kurt-Mothes-Straße 3, 06120 Halle, Germany

^4^ Center for Structural Mass Spectrometry, Martin-Luther-University Halle-Wittenberg, Kurt-Mothes-Straße 3, 06120 Halle, Germany

^5^ Comprehensive Cancer Center Central Germany, Leipzig University, Leipzig, Germany

^6^ Institute of Biology, Faculty of Life Sciences, Leipzig University, Talstrasse 33, 04103 Leipzig, Germany

^*^ Corresponding author

**Supplementary Figures**

**
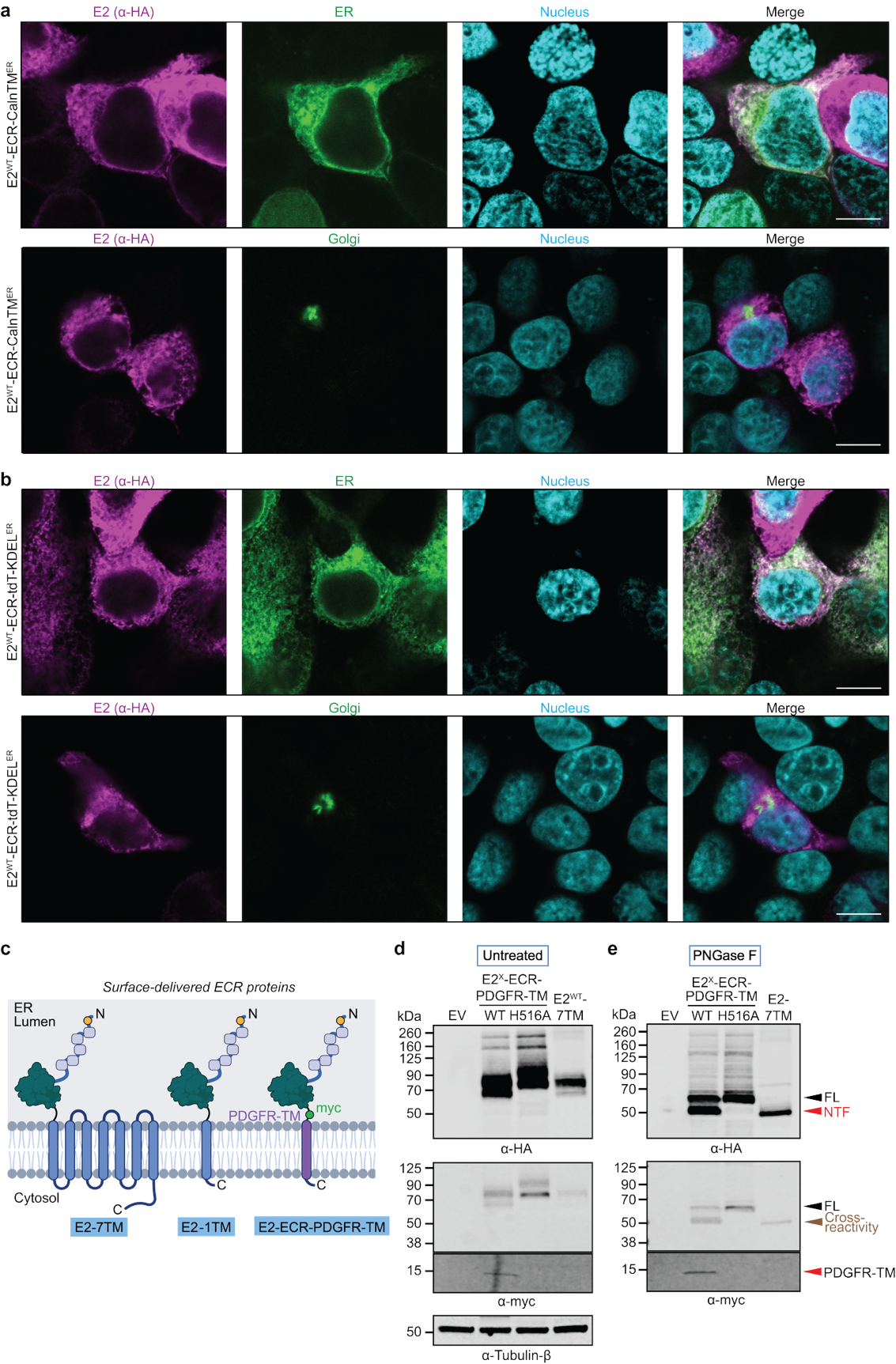
**

**Supplementary Fig. 1, related to Figure 1. TM1 assists with GAIN domain cleavage of E2 via membrane tethering.**

**a-b** HEK293T transiently expressing **(a)** E2^WT^-ECR-CalnTM^ER^, and **(b)** E2^WT^-ECR-tdT-KDEL^ER^ were immunostained against the N-terminal HA tag, followed by confocal microscopy. Localisation of receptor was indicated in purple. ER or Golgi was visualised by CellLight BacMam 2.0 and highlighted in green. Nuclei (Nu) of the cells are stained by Hoechst 33342 and shown in cyan. The brightness and contrast of the images were digitally adjusted to enhance signal visibility, raw images are provided as supplementary files. Scale bar, 10 μm.

**c** Designs of surface-delivered proteins of E2 ECR. The replacement of the TM1 into PDGFR-TM allows efficient targeting of the ECR of E2 (E2-ECR-PDGFR-TM) to the cell surface without any molecular component of the TM regions of the innate E2. Created in BioRender. Chung, Y. (2025) <https://BioRender.com/1svbtwa>.

**d-e** Expression patterns of surface-targeting ECR constructs **(d)** without or **(e)** with PNGase F treatment were verified by Western blotting (using the N-terminal HA tag, and the myc tag in PDGFR-TM). Uncleaved product is indicated in black triangles, while cleaved products, including the NTF and the PDGFR-TM, are shown in red triangles. Unspecific bands from the cross-reactivity of the antibodies were indicated in brown triangle.


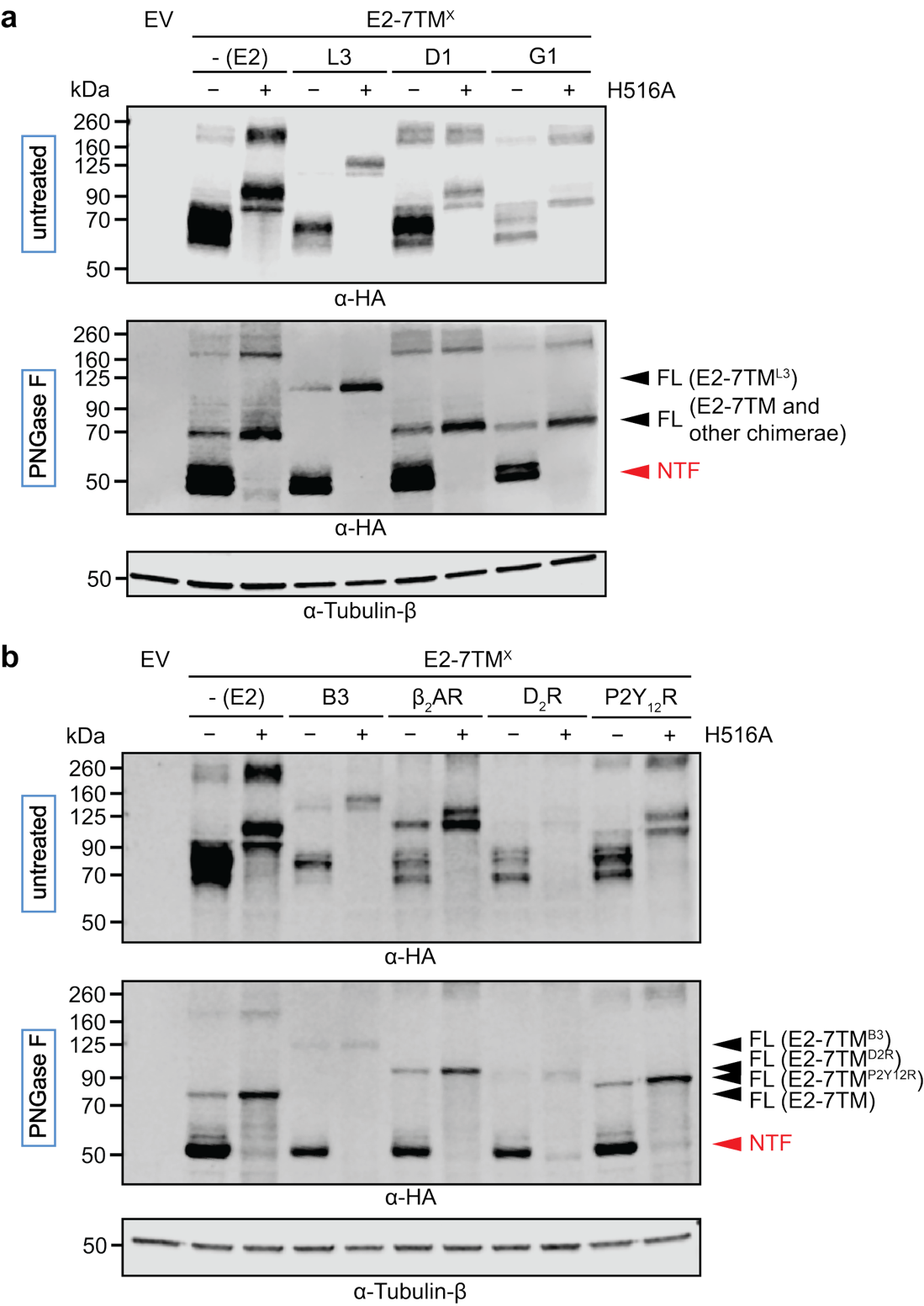


**Supplementary Fig. 2, related to Fig. 2. 7TM region is a physical facilitator of GAIN domain cleavage of E2.**

Representative Western blots of HEK293T expressing E2-ECR conjugated to the 7TM regions of **(a)** cleavable aGPCRs, **(b)** non-cleavable aGPCR (B3) or Class A GPCRs. Lysates were also treated with or without PNGase F. NTF and FL of the receptors are highlighted in red and black triangles, respectively. Open circle, non-specific immunoreactivity. Tubulin-β was also detected as a loading control.


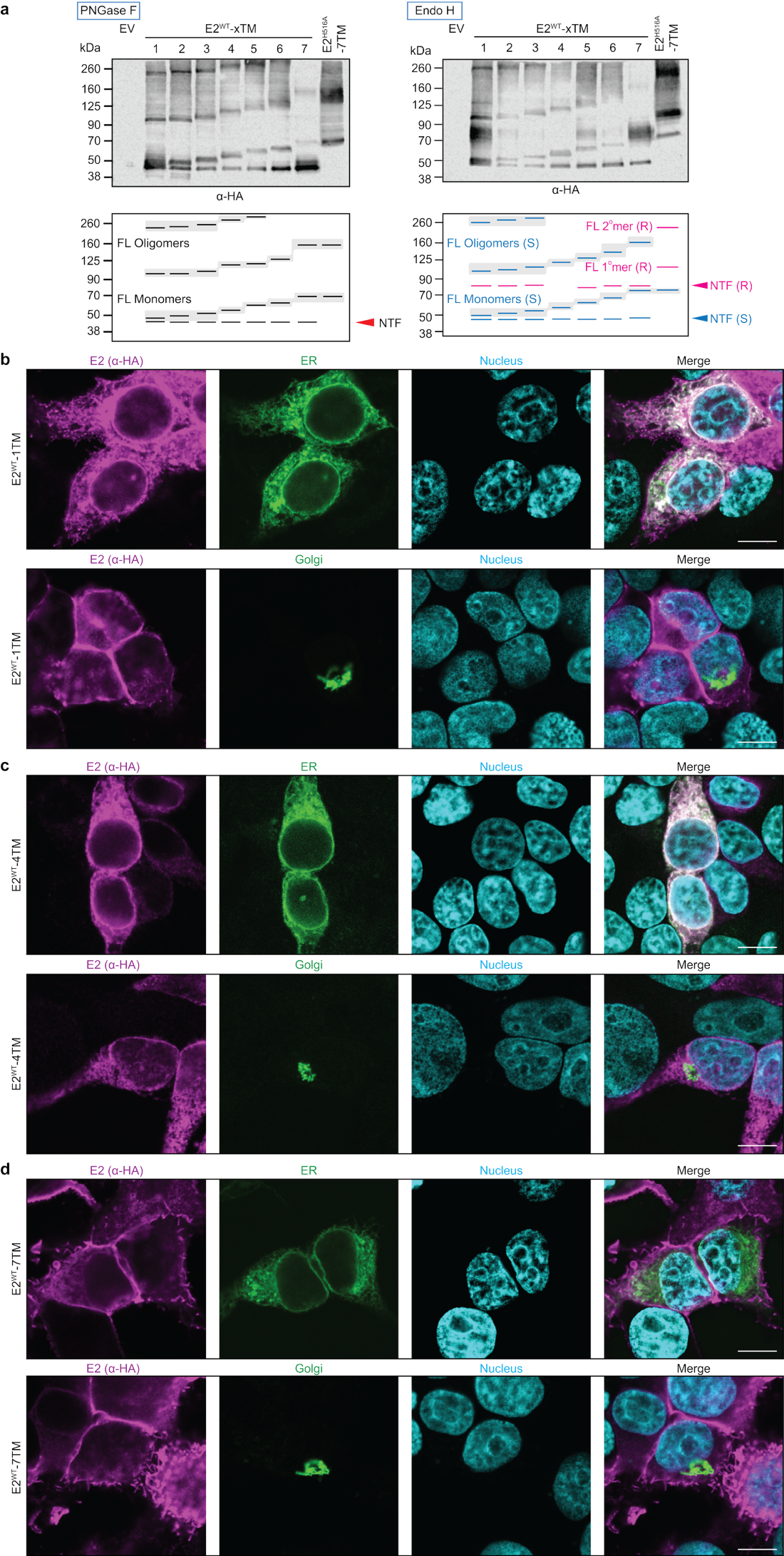


**Supplementary Fig. 3, related to Fig. 3. GAIN domain cleavage promotes ER exit of E2.**

**a** Side-by-side comparison of the Western blots from PNGase F-treated lysates (Fig. 1f) and Endo H-treated lysates (Fig. 3b). Schematics of the two blots indicating the positions of the bands are also shown. Bands are labelled similarly as explained in the respective figure panels.

**b-d** HEK293T transiently expressing **(b)** E2^WT^-1TM, **(c)** E2^WT^-4TM, and **(d)** E2^WT^-7TM were immunostained against the N-terminal HA tag, followed by confocal microscopy. Localisation of receptor was indicated in purple. ER or Golgi was visualised by CellLight BacMam 2.0 and highlighted in green. Nuclei (Nu) of the cells are stained by Hoechst 33342 and shown in cyan. The brightness and contrast of the images were digitally adjusted to enhance signal visibility, raw images are provided as supplementary files. Scale bar, 10 μm.


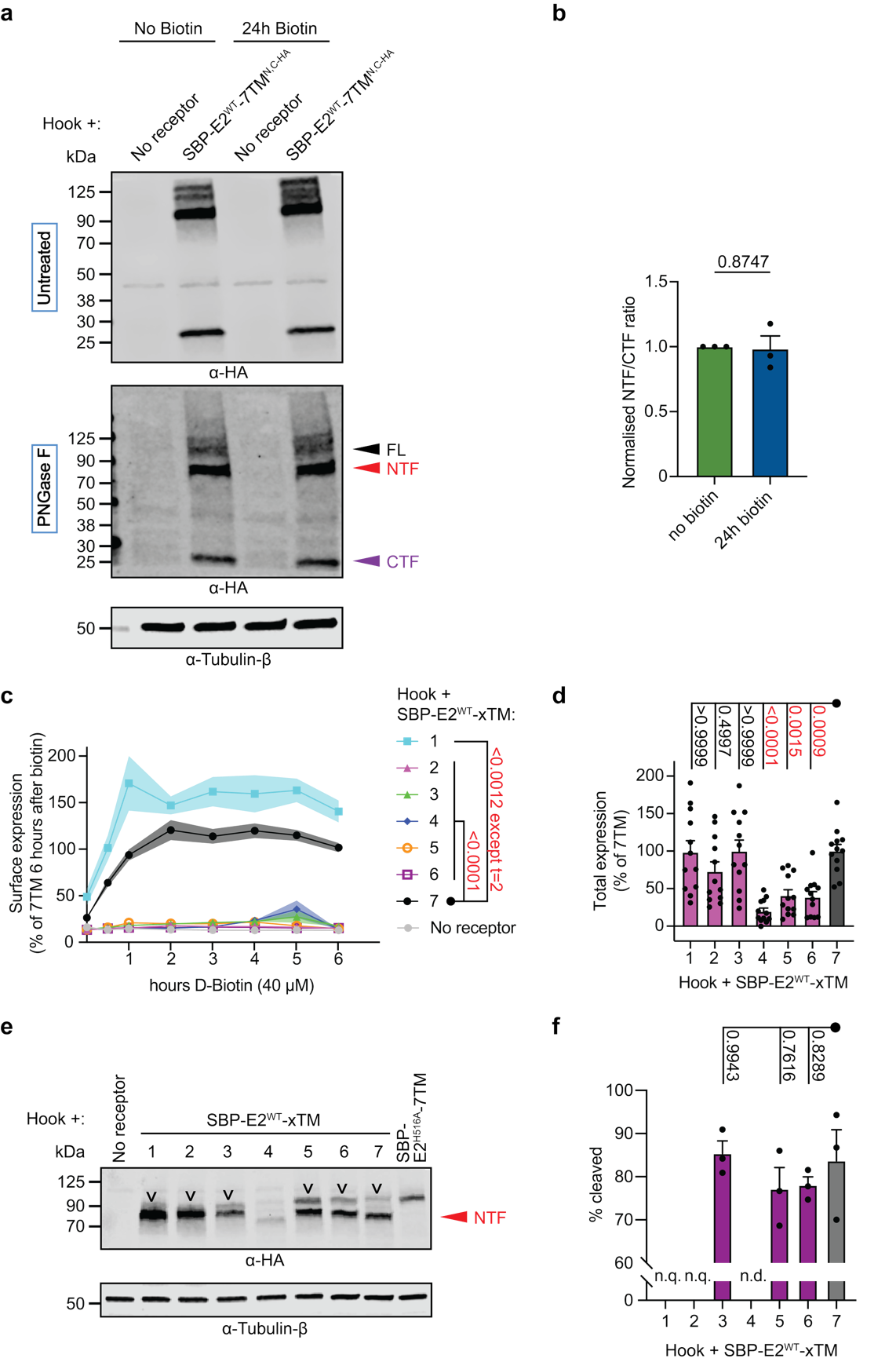


**Supplementary Fig. 4, related to Fig. 3. GAIN domain cleavage promotes ER exit of E2.**

**a-b** SBP-E2^WT^-7TM with HA tags on both N-terminus and the ECL1 of the receptor were transiently expressed in HEK293T cultured in media containing with or without biotin. Representative blot was shown in **(a)**. Lysates were also treated with or without PNGase F. NTF, CTF and FL of the receptors are highlighted in red, purple and black triangles, respectively. Tubulin-β was also detected as a loading control. **b** Quantification on the extent of cleavage was calculated as the ratio of the immunosignal of HA from NTF over that of CTF on blots of lysates under PNGase F treatment, and normalised with the ratio of cells without biotin treatment. Data are shown as mean ± SEM. All individual values are plotted. Data were tested of normality by Shapiro-Wilk test, followed by analysis with unpaired two-tailed t-test (Confidence interval: 95%). P-values are also shown. N=3. Source data are provided as a Source Data file.

**c** HEK293T transiently expressing Hook and SBP-E2^WT^-xTMs were treated with biotin for indicated durations, followed by the detection of surface expressions of SBP-receptors. Data are expressed as the percentage of the signals from SBP-E2^WT^-7TM. Data are shown as mean ± SEM indicated as shaded areas. Data were analysed by two-way ANOVA, followed by Bonferroni’s test with respect to different variants of the same timepoint (Confidence interval: 95%). The p-values for each comparison are listed. N=3, n=4. Source data are provided as a Source Data file.

**d** The cellular expressions of SBP-E2^WT^-xTMs with hook co-expression without biotin treatment were measured by total ELISA. Data are expressed as the percentage of the signals from SBP-E2^WT^-7TM. Data are shown as mean ± SEM. Data were tested of normality by Shapiro-Wilk test, followed by analysis with ordinary one-way ANOVA and Tukey’s test (Confidence interval: 95%). The p-values for each comparison are listed. N=3, n=4. Source data are provided as a Source Data file.

**e-f** SBP-E2^WT^-xTMs were transiently expressed in HEK293T. Representative blot was shown in **(e)**. NTF was highlighted in red triangle, while bands indicative of uncleaved subpopulations are pointed as an inverted caret (_v_). Tubulin-β was also detected as a loading control. **f** Quantification on the extent of cleavage was shown. SBP-E2^WT^-1TM and SBP-E2^WT^-2TM were not quantifiable (n.q.) owing to the failure in resolutions between the NTF and the FL, while SBP-E2^WT^-4TM was undetactable (n.d.). Data are shown as mean ± SEM. All individual values are plotted. Data were tested of normality by Shapiro-Wilk test, followed by analysis with ordinary one-way ANOVA and Tukey’s test (Confidence interval: 95%). P-values are also shown. N=3. Source data are provided as a Source Data file.


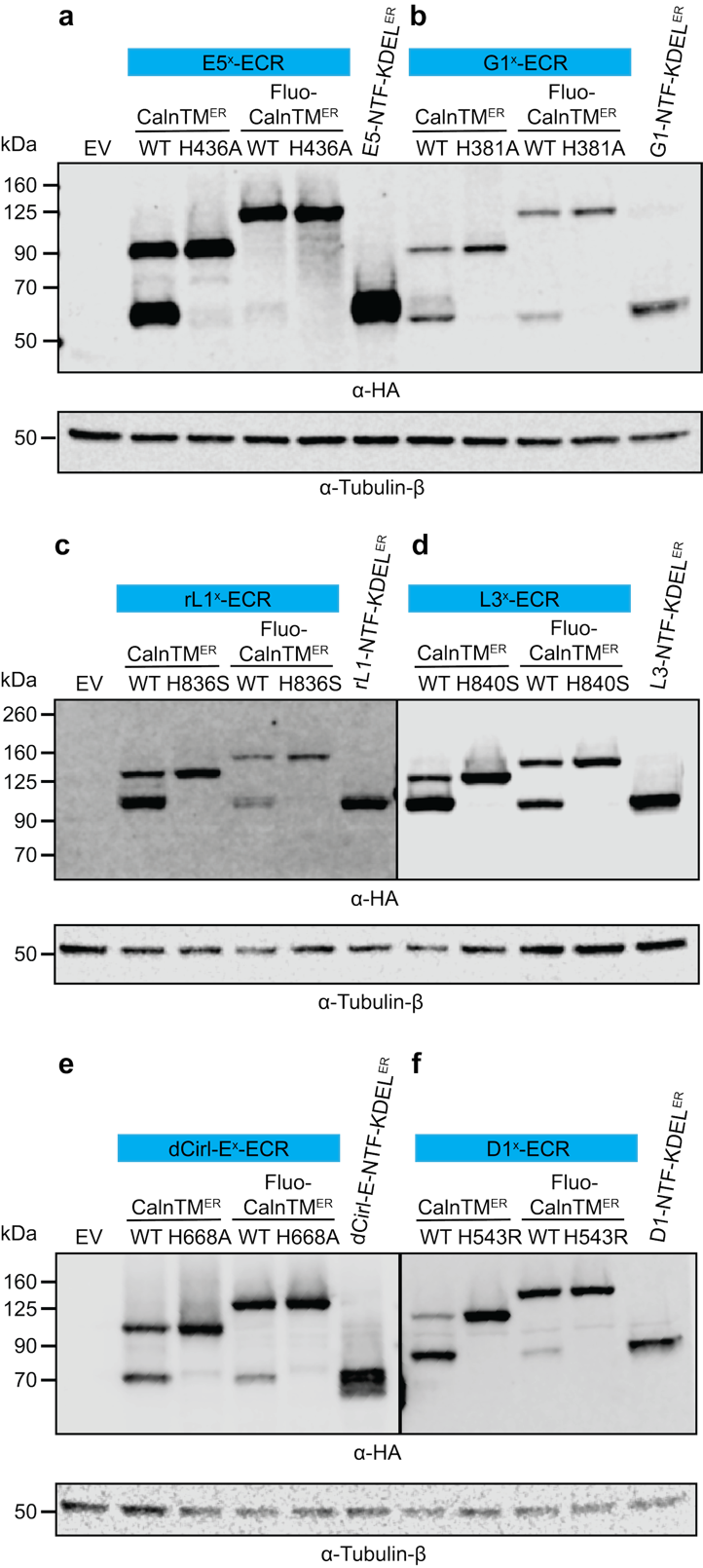


**Supplementary Fig. 5, related to Fig. 4. Membrane proximity is important for GAIN domain cleavage of aGPCRs.**

Representative Western blots showing the expression patterns of ER-anchored constructs of ECRs of different aGPCRs, detected against the N-terminal HA tag. These include **(a)** human ADGRE5/CD97, **(b)** human ADGRG1/GPR56, **(c)** rat ADGRL1/CIRL1/LPHN1, **(d)** human ADGRL3/CIRL3/LPHN3, **(e)** *Dropsophila* homologue of Cirl, isoform type E, and **(f)** human ADGRD1/GPR133. Detection of tubulin-β is also shown as a loading control. Quantification of the GAIN domain cleavabilities of the constructs was shown in Fig. 4c.


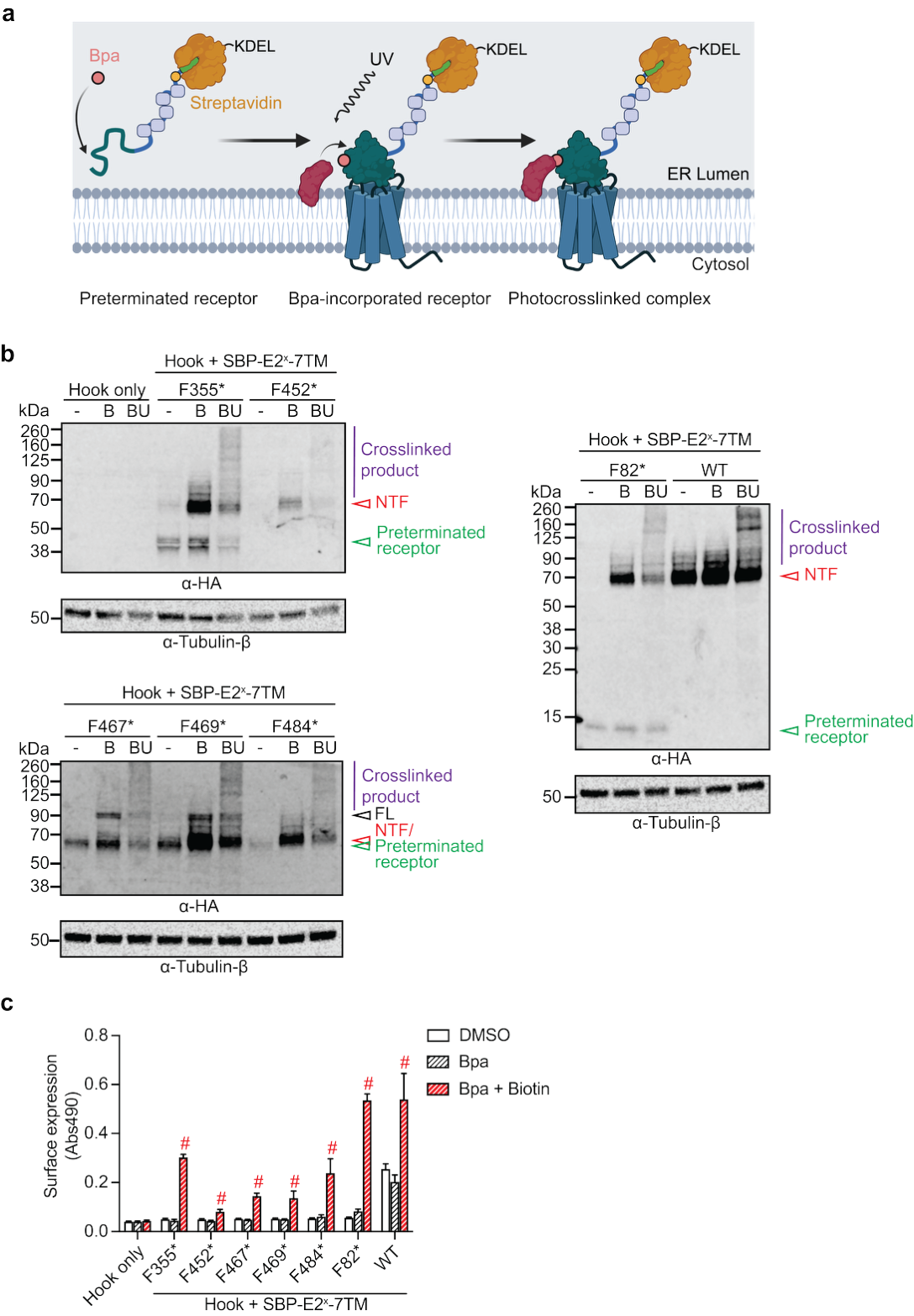


**Supplementary Fig. 6, related to Figure 5. Selection of GAIN domain-specific photosensitive E2-7TM mutant for proteomic analyses.**

**a** The synthesised SBP-receptor is devoid of surface delivery by the ER hook, similarly to the RUSH assay in Fig. 3g. The insertion of the TAG stop codon sequence in the cDNA for incorporation of the unnatural amino acid Bpa results in preterminated synthesis of the protein. Incorporation of Bpa supplemented into culture media on cells co-expressing the transgene and the Bpa-specific tRNA synthetase leads to continuation of translation yielding a receptor containing the photoreactive amino acid. Candidate proteins proximal to the Bpa site will be crosslinked by UV irradiation. Created in BioRender. Chung, Y. (2025) <https://BioRender.com/bgaaqik>.

**b** Expression of SBP-E2-7TM bearing phenylalanine mutations into TAG sequence (marked by asterisk) was verified by Western blotting, detected against the N-terminal HA tag. Cells were treated without (Lanes ‘-’), with Bpa (Lanes ‘B’) or Bpa and UV-irradiated (Lanes ‘BU’) before Lysis. Bands indicative of NTF and FL are shown in red open and black open triangles, respectively. Crosslinked products are indicated in purple. Preterminated receptors are highlighted in green. Detection of tubulin-β is also shown as a loading control.

**c** HEK293T transiently expressing the ER hook and SBP-E2-7TM with or without the TAG mutations was treated with or without Bpa, or Bpa and biotin to release synthesised receptors from the ER. Surface expressions of the receptors were verified by ELISA. Data are shown as mean ± SD. Data were tested of normality by Shapiro-Wilk test, followed by analysis with one-way ANOVA and Tukey’s test (Confidence interval: 95%). #, significantly higher than DMSO-treated samples. N=1, n=4. Source data are provided as a Source Data file.


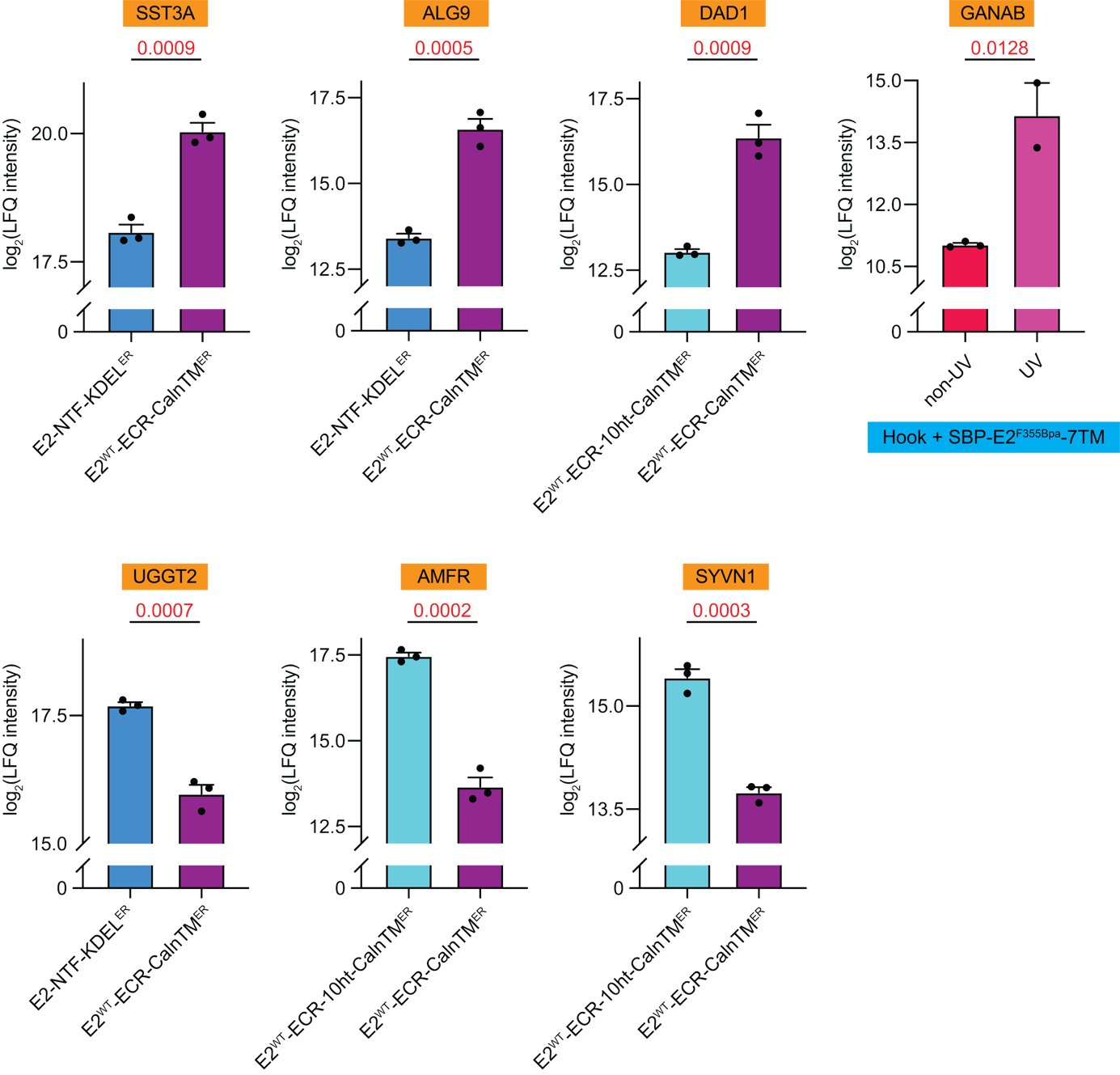


**Supplementary Fig. 7, related to Fig. 5. Proteomic detection of N-glycosylation-related proteins.**

The fold of the LFQ intensity of the proteins pulled down by ER-anchored ECR constructs of E2, or upon UV irradiation on SBP-E2^F355Bpa^-7TM (in the case of GANAB/GIIα) are shown. Data are shown as mean ± SEM. Data were tested of normality by Shapiro-Wilk test, followed by analysis with unpaired two-tailed t-test (Confidence interval: 95%). The p-values are also listed. N=3 except for SBP-E2^F355Bpa^-7TM with UV irradiation where one datapoint was excluded. Source data are provided as a Source Data file.

ALG9 is a mannosyltransferase involved in the formation of the lipid-linked core oligosaccharide GlcNac_2_-Man_9_-Glc_3_ for attachment onto the nascent peptide^56^. SST3A and SST3B are catalytic subunits of the oligosaccharyltransferase (OST) complex that transfers GlcNac_2_-Man_9_-Glc_3_ to target protein^57,58^. DAD1 is a cofactor of the OST complex required for the *N*-glycan transfer^58–60^. GIIα and UGGT are antagonistically involved in the calnexin/calreticulin cycle to ensure proper folding of the synthesised proteins, while misfolded proteins induce ER stress and are redirected to ERAD or UPR^61,62^. GIIα, associated with GIIβ in a heterodimer, acts to remove two inner α-1,3-linked glucoses of the ER-translocating nascent peptide to yield a GlcNAc_2_-Man_9_-linked peptide, and consequently released into the ER lumen from chaperones including calnexin and calreticulin^63–65^. Coincidentally, genetic lesions of either one the GII subunits lead to autosomal-dominant polycystic liver/kidney disease^66–69^, primarily due to the reduction of PC1 GAIN domain cleavage and subsequent curbed ER exit^69^.


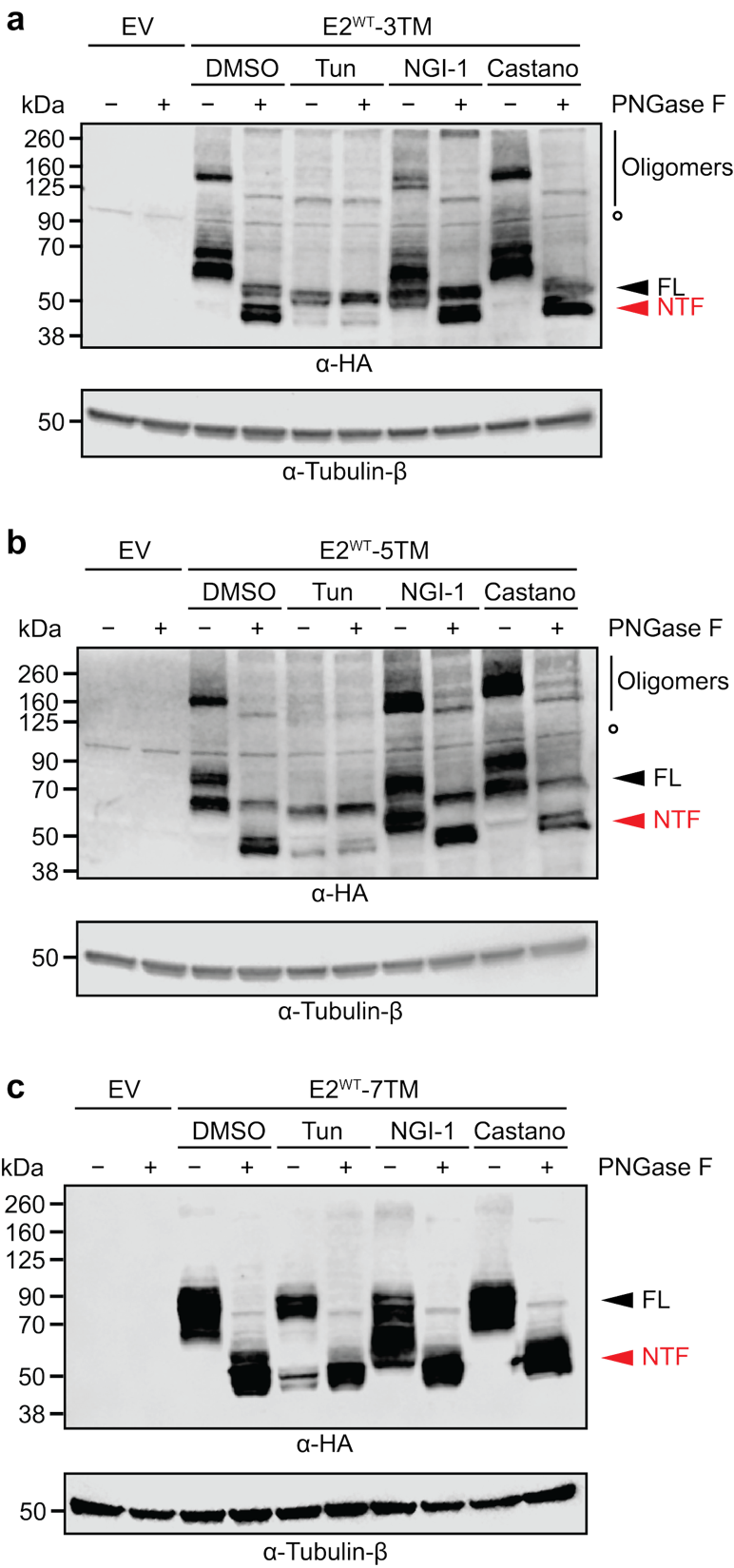


**Supplementary Fig. 8, related to Figure 5. 7TM region compensates for the effect of N-glycosylation on GAIN domain cleavage of E2.**

Representative Western blots of HEK293T expressing **(a)** E2^WT^-3TM, **(b)** E2^WT^-5TM, or **(c)** E2^WT^-7TM, which were subjected to treatments with Tun (10 ng/mL), NGI-1 (10 μM) or Castano (100 μg/mL) for an additional 24 hours before lysis. against different steps of *N*-glycosylation. Lysates were also treated with or without PNGase F. NTF and FL of the receptors are highlighted in red and black triangles, respectively. Open circle, non-specific immunoreactivity. Tubulin-β was also detected as a loading control.

**
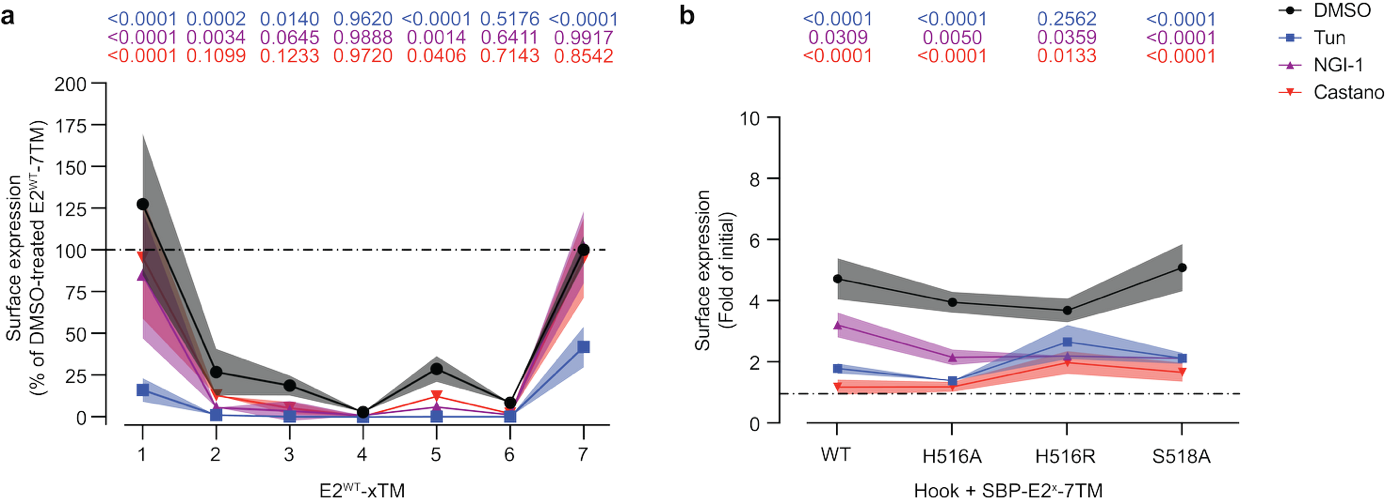
**

**Supplementary Fig. 9, related to Figure 5. Inhibition on N-glycosylation suppresses surface delivery of E2.**

HEK293T cells transiently expressing **(a)** E2^WT^-xTMs, or **(b)** ER hook and SBP-E2-7TM with or without GPS mutations for 24 hours were subjected to treatments with Tun (10 ng/mL), NGI-1 (10 μM) or Castano (100 μg/mL) for an additional 24 hours before detection of surface expressions by ELISA.

**a** Data are expressed as the fraction of the signals from E2^WT^-7TM with DMSO treatment. Data are shown as mean ± SEM indicated in shaded area. The p-values for each comparison are listed following the colour codes of the drug treatments. N=3, n=4. Source data are provided as a Source Data file.

**b** Surface delivery was induced by 3-hour treatment of biotin (40 μM). Data are expressed as the fold of the initial signals of the variants under the same drug treatment. Data are shown as mean ± SEM indicated in shaded area. The p-values for each comparison are listed following the colour codes of the drug treatments. N=3, n=3-4. Source data are provided as a Source Data file.

**Supplementary Tables**

**Supplementary Table 1. Predicted molecular weights of full-length E2^WT^-xTM.**

The molecular weights were predicted by the ExPASy server^70^ based on the primary sequence of the constructs. Post-translational modifications, such as glycosylations, were not considered.

| **Receptor** | **Molecular weight of non-proteolysed full-length protein (kDa)** |
| --- | --- |
| E2-NTF | 57.6 |
| E2^WT^-1TM | 62.9 |
| E2^WT^-2TM | 66.4 |
| E2^WT^-3TM | 70.7 |
| E2^WT^-4TM | 74.9 |
| E2^WT^-5TM | 80.3 |
| E2^WT^-6TM | 84.3 |
| E2^WT^-7TM | 91.6 |

**Supplementary Table 2. List of identified proteins in proteomic analyses of ER-lumenal ECR constructs of E2.**

Proteins are further catagorised into either a lower or higher likelihood of co-immunoprecipitation with E2^WT^-ECR-CalnTM^ER^ bait than the listed bait. ER-resident proteins are highlighted in red.

| **Co-IP bait** | **Lower binding in E2^WT^-ECR-CalnTM^ER^** | **Higher binding in E2^WT^-ECR-CalnTM^ER^** |
| --- | --- | --- |
| **E2-NTF-KDEL^ER^** | \| SORT1 \| GUSB \| TSPYL1 \| CBWD2 \| MED23 \| \| --- \| --- \| --- \| --- \| --- \| \| PANK4 \| UGGT2* \| FOXRED2 \| PPP6R3 \| SFXN3 \| \| PPP6R3 \| CAD \|  \|  \|  \| | \| GPAA1 \| TMEM9 \| LMAN2L \| TOR1AIP2 \| KLF10 \| \| --- \| --- \| --- \| --- \| --- \| \| LAPTM4A \| PIGK \| CHRNA5 \| PIGT \| FAM3A \| \| WFS1 \| PIGU \| RTN3 \| TMTC3 \| ALG9** \| \| DNAJC16 \| MFSD8 \| TMEM43 \| ATP1B3 \| OGFOD3 \| \| SLC38A1 \| ADPGK \| TGFBR1 \| TMEM181 \| LMF1 \| \| GHITM \| UGT8 \| TM9SF2 \| SIGMAR1 \| CLCC1 \| \| TMX1 \| STT3A** \| ERMP1 \| PIGS \| LMBR1 \| \| TMEM214 \| SLC47A1 \| POMT2 \| FAM69B \|  \| |
| **E2^WT^-ECR-10ht-CalnTM^ER^** | \| HIST3H2BB \| DYNC2H1 \| CDS1 \| AMFR* \| SYVN1* \| \| --- \| --- \| --- \| --- \| --- \| \| NDC1 \| XPO4 \| HERPUD1 \| TBC1D15 \| CSE1L \| \| TRAFD1 \| FKBP10 \| NDFIP2 \| ADIPOR1 \| ATP5H \| \| P4HA1 \| TNPO2 \| MTOR \| CAMLG \| GBF1 \| \| TNPO3 \| IPO5 \| TNPO1 \| FHL1 \| TBCD \| \| KIAA1524 \| TJP2 \|  \|  \|  \| | \| COL14A1 \| USMG5 \| KIAA1522 \| NDUFB9 \| DAD1** \| \| --- \| --- \| --- \| --- \| --- \| \| TM9SF1 \| AP3S1 \| TMEM126B \| CCNB1 \| MAST3 \| \| SLC38A1 \| RPS19BP1 \| RAB7A \| SP2 \| RPS28 \| \| EEFSEC \| FEN1 \| LAPTM4A \|  \|  \| |
| **E2^H516A^-ECR-CalnTM^ER^** | None | None |

**Supplementary Table 3. List of identified proteins in Bpa-based proteomic analyses.**

Proteins are further catagorised whether the UV irradiation leads to a significant increase in the crosslinking with SBP-E2^F355Bpa^-7TM. ER-resident protein is highlighted in red.

| **Log_2_-fold change upon UV** | **SBP-E2^F355Bpa^-7TM** |
| --- | --- |
| **>1** | \| PYGL \| HADHB \| EIF4A1 \| RPL30 \| \| --- \| --- \| --- \| --- \| \| MCM6 \| GNB2L1 \| RPSA \| EIF2D \| \| TLN1 \| CHORDC1 \| NUP155 \| ELAC2 \| \| GANAB \| NAA15 \| SLIRP \| AIFM1 \| |
| **<1** | \| CAPZA1 \| WDR6 \| RBM26 \| CPSF4 \| \| --- \| --- \| --- \| --- \| \| UPF3B \| ATAD3A \| HOXB6 \| VANGL2 \| \| EED \| LUC7L3 \| MARK1 \| SETD2 \| \| CGGBP1 \| POLRMT \| SCAF4 \| JAK1 \| \| CBX6 \| RBM8A \| RBM23 \| WDR33 \| \| GTF2H2C \| SCAF11 \| NUMB \| NUDT21 \| \| PPIL2 \| CAAP1 \| RBM27 \| CPSF6 \| \| CCDC8 \| RAB11FIP5 \| DDX28 \| IRS4 \| \| MPG \| FIP1L1 \| PLCD3 \| TBC1D10B \| \| GTPBP2 \| KIF2A \|  \|  \| |

**Supplementary Table 4. Gene Ontology enrichment analysis of ER candidate proteins.**

10 pathways with the highest strength of enrichment are listed and sorted descendingly. Strength is directly proportional to the ratio of observed/background gene count.

| **Pathway description** | **Observed/ Background gene count** | **False discovery rate (×10^-5^)** | **Matching proteins** |
| --- | --- | --- | --- |
| Attachment of GPI anchor to protein | 5/6 | 9.11×10^-8^ | PIGU, PIGT, PIGS, PIGK, GPAA1 |
| ER mannose trimming | 3/14 | 0.0019 | AMFR, UGGT2, SYVN1 |
| Negative regulation of ER stress-induced intrinsic apoptotic signaling pathway | 3/21 | 0.0044 | WFS1, SYVN1, HERPUD1 |
| Protein *N*-linked glycosylation via asparagine | 3/23 | 0.0053 | DAD1, UGGT2, STT3A |
| Glycolipid biosynthetic process | 6/72 | 1.72×10^-5^ | PIGU, PIGT, PIGS, PIGK, GPAA1, UGT8 |
| ER unfolded protein response | 4/54 | 0.0019 | WFS1, AMFR, ERMP1, HERPUD1 |
| ERAD pathway | 6/100 | 5.43×10^-5^ | WFS1, AMFR, UGGT2, SYVN1, FOXRED2, HERPUD1 |
| Ubiquitin-dependent ERAD pathway | 5/82 | 0.00039 | WFS1, AMFR, SYVN1, FOXRED2, HERPUD1 |
| Protein *N*-linked glycosylation | 4/73 | 0.0045 | DAD1, UGGT2, STT3A, ALG9 |
| Cellular response to topologically incorrect protein | 5/99 | 0.00083 | WFS1, AMFR, ERMP1, HERPUD1, UGGT2 |

**Supplementary Table 5. KEGG Pathway analysis of ER candidate proteins.**

Enriched pathways are sorted descendingly according to the strength of enrichment.

| **Pathway description** | **Observed/ Background gene count** | **False discovery rate** | **Matching proteins** |
| --- | --- | --- | --- |
| GPI anchor biosynthesis | 5/24 | 2.92×10^-7^ | PIGU, PIGT, PIGS, PIGK, GPAA1 |
| *N*-glycan biosynthesis | 3/49 | 0.0097 | DAD1, GANAB, STT3A |
| Protein processing in ER | 8/163 | 2.23×10^-7^ | WFS1, DAD1, AMFR, GANAB, UGGT2, SYVN1, STT3A, HERPUD1 |
| Metabolic pathways | 13/1435 | 8.85×10^-5^ | PIGU, DAD1, POMT2, P4HA1, PIGT, CDS1, PIGS, UGT8, ADPGK, GANAB, GPAA1, PIGK, STT3A |

**Supplementary Table 6. List of plasmids used in this study.**

Site-directed mutagenesis was performed using *PfuUltra* High-Fidelity DNA polymerase (Agilent). PCR amplifications of fragments was made using Q5 High-Fidelity DNA polymerase (NEB). Ligations were done by either T4 ligase (Thermofisher) or sequence-ligation-independent cloning using T4 polymerase (NEB). Reactions were designed following manufacturers’ instructions. Unless otherwise specified, all constructs were in pcDNA3.1 vector. The numbering of the mutations follows that of the canonical sequence of the receptor under the Uniprot entry, except E5[125] and dCirlE in which specific isoforms have already been specified. The actual isoform used is indicated in the description section. All plasmids were confirmed by sequencing before downstream applications.

| **Receptor construct** | **Plasmid ID** | **Descriptions** | **Tags** | **Construction method** | **Primers used** |
| --- | --- | --- | --- | --- | --- |
| pcDNA3.1 | pSA25 | Empty pcDNA3.1 vector | - | - | - |
| E2^WT^-7TM | pIB8 | Overexpression of 7TM variant of human ADGRE2/EMR2 in pcDNA3.1 (Uniprot: Q9UHX3-1) | HA | - | - |
| E2^H516A^-7TM | pAC7 | GPS-mutated, cleavage-deficient (H>A) E2-7TM | HA | Ligations of two fragments of 1kb and 1.8kb from pIB8 with 5.3kb fragment of pSA25 (HindIII/XbaI) | 1kb fragment: ib_45F and ac_2R;  1.8kb fragment: ac_1F and ib_46R |
| E2^WT^-TGA | pSA168 | pIB8 with the stop codon of E2 cDNA replaced from TAG to TGA | HA | - | - |
| E2^WT^-1TM | pAC128 | E2 with premature stop codon in the middle of ICL1 | HA | Ligation of two fragments of 1.9kb and 0.8kb from pIB8 with 5.3kb fragment of pSA168 (HindIII/XbaI) | 1.9kb fragment: ac_1F and ac_90R;  0.8kb fragment: ac_89F and ac_2R |
| E2^WT^-2TM | pAC129 | E2 with premature stop codon in the middle of ECL1 | HA | Ligation of two fragments of 2kb and 0.7kb from pIB8 with 5.3kb fragment of pSA168 (HindIII/XbaI) | 1.9kb fragment: ac_1F and ac_143R;  0.8kb fragment: ac_142F and ac_2R |
| E2^WT^-3TM | pAC130 | E2 with premature stop codon in the middle of ICL2 | HA | Ligation of two fragments of 2.1kb and 0.6kb from pIB8 with 5.3kb fragment of pSA168 (HindIII/XbaI) | 1.9kb fragment: ac_1F and ac_150R;  0.8kb fragment: ac_89F and ac_149R |
| E2^WT^-4TM | pAC131 | E2 with premature stop codon in the middle of ECL2 | HA | Ligation of two fragments of 2.2kb and 0.5kb from pIB8 with 5.3kb fragment of pSA168 (HindIII/XbaI) | 1.9kb fragment: ac_1F and ac_152R;  0.8kb fragment: ac_151F and ac_2R |
| E2^WT^-5TM | pAC132 | E2 with premature stop codon in the middle of ICL3 | HA | Ligation of two fragments of 2.3kb and 0.4kb from pIB8 with 5.3kb fragment of pSA168 (HindIII/XbaI) | 1.9kb fragment: ac_1F and ac_154R;  0.8kb fragment: ac_153F and ac_2R |
| E2^WT^-6TM | pAC133 | E2 with premature stop codon in the middle of ECL3 | HA | Ligation of two fragments of 2.5kb and 0.2kb from pIB8 with 5.3kb fragment of pSA168 (HindIII/XbaI) | 1.9kb fragment: ac_1F and ac_156R;  0.8kb fragment: ac_155F and ac_2R |
| Fluo-CalnTM^ER^ | pAC172 | mEmerald attached to the lumenal side of ER membrane by conjugation with calnexin transmembrane region | - | Gift from Ke Xu (Addgene plasmid # 186961) | - |
| E2^WT^-ECR- CalnTM^ER^ | pAC233 | ECR of E2 attached to the lumenal side of ER membrane by conjugation with calnexin transmembrane region | HA | Ligation of a 0.4kb fragment from pAC172 with 7.1kb fragment from pAC164 (AgeI/ApaI) | 0.4kb fragment: ac_249F and ac_2R |
| E2^H516A^-ECR- CalnTM^ER^ | pAC235 | ECR of E2 containing GPS-mutated, cleavage-deficient (H>A) mutation, attached to the lumenal side of ER membrane by conjugation with calnexin transmembrane region | HA | Ligation of a 1.8kb fragment from pAC236 (EcoRI/AgeI) with 5.7kb fragment from pAC233 (EcoRI/AgeI) | - |
| tdT-KDEL^ER^ | pAC135 | Overexpression of ER lumen-localised dimeric TdTomato | - | Gift from Michael Davidson (Addgene plasmid # 58097) | - |
| E2^WT^-ECR-tdT-KDEL^ER^ | pAC164 | ECR of E2 localised in ER lumen by Calreticulin signal sequence and dimeric TdTomato with C-terminal KDEL | HA | Ligation of two fragments of 1.8kb from pIB8 and 1.5kb from pAC135 with 5.3kb of pSA25 (BamHI/EcoRI) | 1.8kb fragment: ac_1F and ac_182R;  1.5kb fragment: ac_180F and ac_181R |
| E2^H516A^-ECR-tdT-KDEL^ER^ | pAC171 | ECR of E2 containing GPS-mutated, cleavage-deficient (H>A) mutation, localised in ER lumen by Calreticulin signal sequence and dimeric TdTomato with C-terminal KDEL | HA | Ligation of two fragments of 1.5kb and 1.8kb from pAC164 with 5.3kb of pSA25 (HindIII/XbaI) | 1.5kb fragment: ib_45F and ac_2R;  1.8kb fragment: ac_1F and ib_46R |
| E2-NTF-KDEL^ER^ | pAC166 | NTF of E2 with a C-terminal KDEL | HA | Ligation of 1.6kb fragment from pIB8 with 5.3kb of pSA25 (HindIII/XbaI) | 1.6kb fragment: ac_1F and ac_188R |
| pDisplay | pAC159 | Empty pDisplay vector | - | - | - |
| E2^WT^-ECR-PDGFR-TM | pAC170 | ECR of E2, followed by PDGFR transmembrane region | HA/myc | Ligation of 1.6kb fragment from pIB8 with 5.3kb fragment of pAC159 (ApaI/SalI) | 1.6kb fragment: ac_197F and ac_198R |
| E2^H516A^-ECR-PDGFR-TM | pAC239 | ECR of E2 containing GPS-mutated, cleavage-deficient (H>A) mutation, followed by PDGFR transmembrane region | HA/myc | Ligation of 1.6kb fragment from pAC7 with 5.3kb fragment of pAC159 (ApaI/SalI) | 1.6kb fragment: ac_197F and ac_198R |
| E2^WT^-7TM^L3^ | pAC396 | ECR of E2 followed by the 7TM region of L3 | HA | Ligation of two 1.8kb and 1.8 kb fragments from pIB8 and pIB69 (human L3 in pcDNA3.1) respectively with 5.3kb fragment of pSA25 (HindIII/XbaI) | 1.8kb fragment from pIB8: ac_1F and ac_337R  1.8kb fragment from pIB69: ac_336F and ac_66R |
| E2^H516A^-7TM^L3^ | pAC397 | ECR of E2 containing GPS-mutated, cleavage-dficient (H>A) mutation, followed by the 7TM region of L3 | HA | Ligation of two 1.8kb and 1.8 kb fragments from pAC7 and pIB69 respectively with 5.3kb fragment of pSA25 (HindIII/XbaI) | 1.8kb fragment from pIB8: ac_1F and ac_337R  1.8kb fragment from pIB69: ac_336F and ac_66R |
| E2^WT^-7TM^D1^ | pAC392 | ECR of E2 followed by the 7TM region of D1 | HA | Ligation of two 1.8kb and 1 kb fragments from pIB8 and pMIH020 (human D1 in pcDNA3.1) respectively with 5.3kb fragment of pSA25 (HindIII/XbaI) | 1.8kb fragment: ac_1F and ac_337R  1kb fragment: ac_338F and ac_21R |
| E2^H516A^-7TM^D1^ | pAC393 | ECR of E2 containing GPS-mutated, cleavage-dficient (H>A) mutation, followed by the 7TM region of D1 | HA | Ligation of two 1.8kb and 1 kb fragments from pAC7 and pMIH020 respectively with 5.3kb fragment of pSA25 (HindIII/XbaI) | 1.8kb fragment: ac_1F and ac_337R  1kb fragment: ac_338F and ac_21R |
| E2^WT^-7TM^G1^ | pAC394 | ECR of E2 followed by the 7TM region of G1 | HA | Ligation of two 1.8kb and 1 kb fragments from pIB8 and pAC31 (human G1 in pcDNA3.1) respectively with 5.3kb fragment of pSA25 (HindIII/XbaI) | 1.8kb fragment: ac_1F and ac_337R  1kb fragment: ac_339F and ac_21R |
| E2^H516A^-7TM^G1^ | pAC395 | ECR of E2 containing GPS-mutated, cleavage-dficient (H>A) mutation, followed by the 7TM region of G1 | HA | Ligation of two 1.8kb and 1 kb fragments from pAC7 and pAC31 respectively with 5.3kb fragment of pSA25 (HindIII/XbaI) | 1.8kb fragment: ac_1F and ac_337R  1kb fragment: ac_339F and ac_21R |
| E2^WT^-7TM^B3^ | pAC408 | ECR of E2 followed by the 7TM region of B3 | HA | Ligation of two 1.8kb and 2 kb fragments from pIB8 and pNH221 (human B3 in pcDNA3.1) respectively with 5.3kb fragment of pSA25 (HindIII/XbaI) | 1.8kb fragment: ac_1F and ac_337R  2kb fragment: ac_368F and ac_68R |
| E2^H516A^-7TM^B3^ | pAC412 | ECR of E2 containing GPS-mutated, cleavage-dficient (H>A) mutation, followed by the 7TM region of B3 | HA | Ligation of two 1.8kb and 2 kb fragments from pAC7 and pNH221 respectively with 5.3kb fragment of pSA25 (HindIII/XbaI) | 1.8kb fragment: ac_1F and ac_337R  1kb fragment: ac_368F and ac_68R |
| E2^WT^-7TM^β2AR^ | pAC402 | ECR of E2 followed by the 7TM region of β_2_-adrenergic receptor | HA | Ligation of two 1.8kb and 1.2 kb fragments from pIB8 and pAC304 (human β2AR-Nluc-N-ter) respectively with 5.3kb fragment of pSA25 (HindIII/XbaI) | 1.8kb fragment: ac_1F and ac_337R  1.2kb fragment: ac_362F and ac_363R |
| E2^H516A^-7TM^β2AR^ | pAC409 | ECR of E2 containing GPS-mutated, cleavage-dficient (H>A) mutation, followed by the 7TM region of β_2_-adrenergic receptor | HA | Ligation of two 1.8kb and 1.2 kb fragments from pAC7 and pAC304 respectively with 5.3kb fragment of pSA25 (HindIII/XbaI) | 1.8kb fragment: ac_1F and ac_337R  1.2kb fragment: ac_362F and ac_363R |
| E2^WT^-7TM^D2R^ | pAC406 | ECR of E2 followed by the 7TM region of Dopamine D_2_ receptor | HA | Ligation of two 1.8kb and 1.3 kb fragments from pIB8 and pIB48 (human D_2_R in pCAGGS) respectively with 5.3kb fragment of pSA25 (HindIII/XbaI) | 1.8kb fragment: ac_1F and ac_337R  1.2kb fragment: ac_364F and ac_365R |
| E2^H516A^-7TM^D2R^ | pAC410 | ECR of E2 containing GPS-mutated, cleavage-dficient (H>A) mutation, followed by the 7TM region of Dopamine D_2_ receptor | HA | Ligation of two 1.8kb and 1.3 kb fragments from pAC7 and pIB48 respectively with 5.3kb fragment of pSA25 (HindIII/XbaI) | 1.8kb fragment: ac_1F and ac_337R  1.2kb fragment: ac_364F and ac_365R |
| E2^WT^-7TM^P2Y12R^ | pAC407 | ECR of E2 followed by the 7TM region of Purinergic P2Y_12_ receptor | HA | Ligation of two 1.8kb and 1 kb fragments from pIB8 and pSA55 (human P2Y_12_R in pcDps) respectively with 5.3kb fragment of pSA25 (HindIII/XbaI) | 1.8kb fragment: ac_1F and ac_337R  1kb fragment: ac_366F and ac_367R |
| E2^H516A^-7TM^P2Y12R^ | pAC411 | ECR of E2 containing GPS-mutated, cleavage-dficient (H>A) mutation, followed by the 7TM region of Dopamine D_2_ receptor | HA | Ligation of two 1.8kb and 1.3 kb fragments from pAC7 and pSA55 respectively with 5.3kb fragment of pSA25 (HindIII/XbaI) | 1.8kb fragment: ac_1F and ac_337R  1kb fragment: ac_366F and ac_367R |
| E2^S518A^-7TM | pAC8 | GPS-mutated, cleavage-deficient (S>A) E2-7TM | HA | Ligations of two fragments of 1kb and 1.8kb from pIB8 with 5.3kb fragment of pSA25 (HindIII/XbaI) | 1kb fragment: ib_39F and ac_2R;  1.8kb fragment: ac_1F and ib_40R |
| E2^H516R^-7TM | pAC10 | GPS-mutated, cleavage-deficient (H>R) E2-7TM | HA | Ligations two fragments of 1kb and 1.8kb from pIB8 with 5.3kb fragment of pSA25 (HindIII/XbaI) | 1kb fragment: ac_7F and ac_2R;  1.8kb fragment: ac_1F and ac_8R |
| StrpKDEL | pAC2 | Overexpression of the ER-localised streptavidin (Hook) for the RUSH assay | - | Gift from Franck Perez (Addgene No. 65306) |  |
| StrpKDEL_SBP-eGFP-CCR5 | pAC6 | Bicistronic vector expressing ER-localised streptavidin and SBP- and eGFP-conjugated CCR5 | - | Gift from Franck Perez |  |
| StrpKDEL_SBP-eGFP-E2^WT^-7TM | pAC39 | Bicistronic vector expressing ER-localised streptavidin and SBP- and eGFP-conjugated E2^WT^-7TM with its start Methionine and the innate signal peptide removed. The eGFP is positioned between the SBP and the HA tag in the N-terminus of the receptor. | HA | 1. Ligations of two fragments of 1kb and 1.8kb from pIB8 from pIB8 with 5.9kb fragment of pAC6 (BamHI/XbaI) 2. Deleting signal peptide and starting Methionine of the innate E2 CDR by site-directed mutagenesis | 1. 1kb fragment: ac_11F and ac_2R; 1.8kb fragment: ac_9F and ac_10R 2. ac_55F and ac_54R |
| StrpKDEL_SBP-E2^WT^-7TM | pAC50 | Bicistronic vector expressing ER-localised streptavidin and SBP-conjugated E2 | HA | Synthesised by Genscript | - |
| StrpKDEL_SBP-E2^WT^-7TM^N,C-HA^ | pAC54 | Bicistronic vector expressing ER-localised streptavidin and SBP-conjugated E2, with HA tags on N-terminus and ECL1 | HA | Ligation of 1.3kb fragment of pAC25 (E2^WT^-7TM^C-HA^) (BstEII/XbaI) with 6.3kb fragment of pAC50 (BstEII/XbaI) | - |
| StrpKDEL_SBP-E2^H516A^-7TM | pAC72 | Bicistronic vector expressing ER-localised streptavidin and SBP-conjugated E2 containing GPS-mutated, cleavage-deficient (H>A) mutation | HA | Ligation of 1.3kb fragment of pAC7 (BstEII/XbaI) with 6.3kb fragment of pAC50 (BstEII/XbaI) | - |
| StrpKDEL_SBP-E2^S518A^-7TM | pAC73 | Bicistronic vector expressing ER-localised streptavidin and SBP-conjugated E2 containing GPS-mutated, cleavage-deficient (S>A) mutation | HA | Ligation of 1.3kb fragment of pAC8 (BstEII/XbaI) with 6.3kb fragment of pAC50 (BstEII/XbaI) | - |
| StrpKDEL_SBP-E2^H516R^-7TM | pAC74 | Bicistronic vector expressing ER-localised streptavidin and SBP-conjugated E2 containing GPS-mutated, cleavage-deficient (H>R) mutation | HA | Ligation of 1.3kb fragment of pAC10 (BstEII/XbaI) with 6.3kb fragment of pAC50 (BstEII/XbaI) | - |
| StrpKDEL_SBP-E2^WT^-1TM | pAC91 | Bicistronic vector expressing ER-localised streptavidin and SBP-conjugated E2 with premature stop codon in the middle of ICL1 | HA | Ligation of 1.3kb fragment of pAC128 (BstEII/XbaI) with 6.3kb fragment of pAC50 (BstEII/XbaI) | - |
| StrpKDEL_SBP-E2^WT^-2TM | pAC112 | Bicistronic vector expressing ER-localised streptavidin and SBP-conjugated E2 with premature stop codon in the middle of ECL1 | HA | Ligation of 1.3kb fragment of pAC129 (BstEII/XbaI) with 6.3kb fragment of pAC50 (BstEII/XbaI) | - |
| StrpKDEL_SBP-E2^WT^-3TM | pAC137 | Bicistronic vector expressing ER-localised streptavidin and SBP-conjugated E2 with premature stop codon in the middle of ICL2 | HA | Ligation of 1.3kb fragment of pAC130 (BstEII/XbaI) with 6.3kb fragment of pAC50 (BstEII/XbaI) | - |
| StrpKDEL_SBP-E2^WT^-4TM | pAC157 | Bicistronic vector expressing ER-localised streptavidin and SBP-conjugated E2 with premature stop codon in the middle of ECL2 | HA | Ligation of 1.3kb fragment of pAC131 (BstEII/XbaI) with 6.3kb fragment of pAC50 (BstEII/XbaI) | - |
| StrpKDEL_SBP-E2^WT^-5TM | pAC138 | Bicistronic vector expressing ER-localised streptavidin and SBP-conjugated E2 with premature stop codon in the middle of ICL3 | HA | Ligation of 1.3kb fragment of pAC132 (BstEII/XbaI) with 6.3kb fragment of pAC50 (BstEII/XbaI) | - |
| StrpKDEL_SBP-E2^WT^-6TM | pAC158 | Bicistronic vector expressing ER-localised streptavidin and SBP-conjugated E2 with premature stop codon in the middle of ECL3 | HA | Ligation of 1.3kb fragment of pAC133 (BstEII/XbaI) with 6.3kb fragment of pAC50 (BstEII/XbaI) | - |
| E2^WT^-ECR-Fluo-CalnTM^ER^ | pAC234 | ECR of E2 attached to the lumenal side of ER membrane by conjugation with a mEmerald, followed by a calnexin transmembrane region | HA | Ligation of 1kb fragment from pAC172 (AgeI/ApaI) with 7.1kb fragment from pAC164 (AgeI/ApaI) | - |
| E2^H516A^-ECR-Fluo-CalnTM^ER^ | pAC236 | ECR of E2 containing GPS-mutated, cleavage-deficient (H>A) mutation, attached to the lumenal side of ER membrane by conjugation with a mEmerald, followed by a calnexin transmembrane region | HA | Ligation of 1kb fragment from pAC172 (AgeI/ApaI) with 7.1kb fragment from pAC171 (AgeI/ApaI) | - |
| E5^WT^-ECR-CalnTM^ER^ | pAC246 | ECR of E5 attached to the lumenal side of ER membrane by conjugation with calnexin transmembrane region (Uniprot: P48960-2) | HA | Ligation of two fragments of 0.4kb fragment from pAC233 and 1.4kb fragment from pSA26 (E5) with 5.3kb fragment from pSA25 (HindIII/XbaI) | 0.4kb fragment: ac_257F and ac_272R;  1.4kb fragment: ac_1F and ac_258R |
| E5^H436A^-ECR-CalnTM^ER^ | pAC247 | ECR of E5 containing GPS-mutated, cleavage-deficient (H>A) mutation, attached to the lumenal side of ER membrane by conjugation with calnexin transmembrane region | HA | Ligation of two fragments of 0.4kb fragment from pAC233 and 1.4kb fragment from pSA28 (E5^H436A^) with 5.3kb fragment from pSA25 (HindIII/XbaI) | 0.4kb fragment: ac_257F and ac_272R;  1.4kb fragment: ac_1F and ac_258R |
| E5^WT^-ECR-Fluo-CalnTM^ER^ | pAC248 | ECR of E5 attached to the lumenal side of ER membrane by conjugation with a mEmerald, followed by a calnexin transmembrane region | HA | Ligation of two fragments of 1.1kb fragment from pAC234 and 1.4kb fragment from pSA26 (E5) with 5.3kb fragment from pSA25 (HindIII/XbaI) | 1.1kb fragment: ac_257F and ac_272R;  1.4kb fragment: ac_1F and ac_258R |
| E5^H436A^-ECR-Fluo-CalnTM^ER^ | pAC249 | ECR of E5 containing GPS-mutated, cleavage-deficient (H>A) mutation, attached to the lumenal side of ER membrane by conjugation with a mEmerald, followed by a calnexin transmembrane region | HA | Ligation of two fragments of 1.1kb fragment from pAC234 and 1.4kb fragment from pSA28 (E5^H436A^) with 5.3kb fragment from pSA25 (HindIII/XbaI) | 1.1kb fragment: ac_257F and ac_272R;  1.4kb fragment: ac_1F and ac_258R |
| E5-NTF-KDEL^ER^ | pAC250 | NTF of E5 with a C-terminal KDEL | HA | Ligation of 1.4kb fragment from pSA26 (E5) with 5.3kb of pSA25 (HindIII/XbaI) | 1.4kb fragment: ac_1F and ac_261R |
| L3^WT^-ECR-CalnTM^ER^ | pAC251 | ECR of L3 attached to the lumenal side of ER membrane by conjugation with calnexin transmembrane region (Uniprot code: Q9HAR2-4) | HA | Ligation of two fragments of 0.4kb fragment from pAC233 and 2.8kb fragment from pMAK01 (hL3-NRS) with 5.3kb fragment from pSA25 (HindIII/XbaI) | 0.4kb fragment: ac_257F and ac_272R;  2.8kb fragment: ac_1F and ac_260R |
| L3^H840S^-ECR-CalnTM^ER^ | pAC252 | ECR of L3 containing GPS-mutated, cleavage-deficient (H>S) mutation, attached to the lumenal side of ER membrane by conjugation with calnexin transmembrane region | HA | Ligation of two fragments of 0.4kb fragment from pAC233 and 2.8kb fragment from pMAK03 (hL3^H840S^-NRS) with 5.3kb fragment from pSA25 (HindIII/XbaI) | 0.4kb fragment: ac_257F and ac_272R;  2.8kb fragment: ac_1F and ac_260R |
| L3^WT^-ECR-Fluo-CalnTM^ER^ | pAC253 | ECR of L3 attached to the lumenal side of ER membrane by conjugation with a mEmerald, followed by a calnexin transmembrane region | HA | Ligation of two fragments of 1.1kb fragment from pAC234 and 2.8kb fragment from pMAK01 (hL3-NRS) with 5.3kb fragment from pSA25 (HindIII/XbaI) | 1.1kb fragment: ac_257F and ac_272R;  2.8kb fragment: ac_1F and ac_260R |
| L3^H840S^-ECR-Fluo-CalnTM^ER^ | pAC254 | ECR of L3 containing GPS-mutated, cleavage-deficient (H>S) mutation, attached to the lumenal side of ER membrane by conjugation with a mEmerald, followed by a calnexin transmembrane region | HA | Ligation of two fragments of 1.1kb fragment from pAC234 and 2.8kb fragment from pMAK03 (hL3^H840S^-NRS) 5.3kb fragment from pSA25 (HindIII/XbaI) | 1.1kb fragment: ac_257F and ac_272R;  2.8kb fragment: ac_1F and ac_260R |
| L3-NTF-KDEL^WT^ | pAC255 | NTF of L3 with a C-terminal KDEL | HA | Ligation of 2.8kb fragment from pMAK01 (hL3-NRS) with 5.3kb of pSA25 (HindIII/XbaI) | 2.8kb fragment: ac_1F and ac_263R |
| D1^WT^-ECR-CalnTM^ER^ | pAC256 | ECR of D1 attached to the lumenal side of ER membrane by conjugation with calnexin transmembrane region (Uniprot: Q6QNK2-1) | HA | Ligation of two fragments of 0.4kb fragment from pAC233 and 1.8kb fragment from pMIH020 (D1) with 5.3kb fragment from pSA25 (HindIII/XbaI) | 0.4kb fragment: ac_257F and ac_272R;  1.8kb fragment: ac_1F and ac_270R |
| D1^H543R^-ECR-CalnTM^ER^ | pAC257 | ECR of D1 containing GPS-mutated, cleavage-deficient (H>R) mutation, attached to the lumenal side of ER membrane by conjugation with calnexin transmembrane region | HA | Ligation of two fragments of 0.4kb fragment from pAC233 and 1.8kb fragment from pAC49 (D1^H543R^) with 5.3kb fragment from pSA25 (HindIII/XbaI) | 0.4kb fragment: ac_257F and ac_272R;  1.8kb fragment: ac_1F and ac_270R |
| D1^WT^-ECR-Fluo-CalnTM^ER^ | pAC258 | ECR of D1 attached to the lumenal side of ER membrane by conjugation with a mEmerald, followed by a calnexin transmembrane region | HA | Ligation of two fragments of 1.1kb fragment from pAC234 and 1.8kb fragment from pMIH020 (D1) with 5.3kb fragment from pSA25 (HindIII/XbaI) | 1.1kb fragment: ac_257F and ac_272R;  1.8kb fragment: ac_1F and ac_270R |
| D1^H543R^-ECR-Fluo-CalnTM^ER^ | pAC259 | ECR of D1 containing GPS-mutated, cleavage-deficient (H>R) mutation, attached to the lumenal side of ER membrane by conjugation with a mEmerald, followed by a calnexin transmembrane region | HA | Ligation of two fragments of 1.1kb fragment from pAC234 and 1.8kb fragment from pAC49 (D1^H543R^) with 5.3kb fragment from pSA25 (HindIII/XbaI) | 1.1kb fragment: ac_257F and ac_272R;  1.8kb fragment: ac_1F and ac_270R |
| D1-NTF-KDEL^ER^ | pAC260 | NTF of D1 with a C-terminal KDEL | HA | Ligation of 1.8kb fragment from pMIH020 (D1) with 5.3kb of pSA25 (HindIII/XbaI) | 1.8kb fragment: ac_1F and ac_265R |
| rL1^WT^-ECR-CalnTM^ER^ | pAC261 | ECR of L1 attached to the lumenal side of ER membrane by conjugation with calnexin transmembrane region (Uniprot: O88917-2) | HA | Ligation of two fragments of 0.4kb fragment from pAC233 and 2.6kb fragment from pSA132 (L1) with 5.3kb fragment from pSA25 (HindIII/XbaI) | 0.4kb fragment: ac_257F and ac_272R;  2.6kb fragment: ac_1F and ac_259R |
| rL1^H836A^-ECR-CalnTM^ER^ | pAC263 | ECR of L1 containing GPS-mutated, cleavage-deficient (H>A) mutation, attached to the lumenal side of ER membrane by conjugation with calnexin transmembrane region | HA | Ligation of two fragments of 0.4kb fragment from pAC233 and 2.6kb fragment from pSA163 (L1^H836S^) with 5.3kb fragment from pSA25 (HindIII/XbaI) | 0.4kb fragment: ac_257F and ac_272R;  2.6kb fragment: ac_1F and ac_259R |
| rL1^WT^-ECR-Fluo-CalnTM^ER^ | pAC262 | ECR of L1 attached to the lumenal side of ER membrane by conjugation with a mEmerald, followed by a calnexin transmembrane region | HA | Ligation of two fragments of 1.1kb fragment from pAC234 and 2.6kb fragment from pSA132 (L1) with 5.3kb fragment from pSA25 (HindIII/XbaI) | 1.1kb fragment: ac_257F and ac_272R;  2.6kb fragment: ac_1F and ac_259R |
| rL1^H836A^-ECR-Fluo-CalnTM^ER^ | pAC264 | ECR of L1 containing GPS-mutated, cleavage-deficient (H>A) mutation, attached to the lumenal side of ER membrane by conjugation with a mEmerald, followed by a calnexin transmembrane region | HA | Ligation of two fragments of 1.1kb fragment from pAC234 and 2.6kb fragment from pSA163 (L1^H836S^) with 5.3kb fragment from pSA25 (HindIII/XbaI) | 1.1kb fragment: ac_257F and ac_272R;  2.6kb fragment: ac_1F and ac_259R |
| rL1-NTF-KDEL^ER^ | pAC265 | NTF of L1 with a C-terminal KDEL | HA | Ligation of 2.6kb fragment from pSA132 (L1) with 5.3kb of pSA25 (HindIII/XbaI) | 2.6kb fragment: ac_1F and ac_262R |
| G1^WT^-ECR-CalnTM^ER^ | pAC266 | ECR of G1 attached to the lumenal side of ER membrane by conjugation with calnexin transmembrane region (Uniprot: Q9Y653-2) | HA | Ligation of two fragments of 0.4kb fragment from pAC233 and 1.2kb fragment from pAC31 (G1) with 5.3kb fragment from pSA25 (HindIII/XbaI) | 0.4kb fragment: ac_257F and ac_272R; 1.2kb fragment: ac_1F and ac_271R |
| G1^H381A^-ECR-CalnTM^ER^ | pAC268 | ECR of G1 containing GPS-mutated, cleavage-deficient (H>A) mutation, attached to the lumenal side of ER membrane by conjugation with calnexin transmembrane region | HA | Ligation of two fragments of 0.4kb fragment from pAC233 and 1.2kb fragment from pAC40 (G1^H381A^) with 5.3kb fragment from pSA25 (HindIII/XbaI) | 0.4kb fragment: ac_257F and ac_272R; 1.2kb fragment: ac_1F and ac_271R |
| G1^WT^-ECR-Fluo-CalnTM^ER^ | pAC267 | ECR of G1 attached to the lumenal side of ER membrane by conjugation with a mEmerald, followed by a calnexin transmembrane region | HA | Ligation of two fragments of 1.1kb fragment from pAC234 and 1.2kb fragment from pAC31 (G1) with 5.3kb fragment from pSA25 (HindIII/XbaI) | 1.1kb fragment: ac_257F and ac_272R; 1.2kb fragment: ac_1F and ac_271R |
| G1^H381A^-ECR-Fluo-CalnTM^ER^ | pAC269 | ECR of G1 containing GPS-mutated, cleavage-deficient (H>A) mutation, attached to the lumenal side of ER membrane by conjugation with a mEmerald, followed by a calnexin transmembrane region | HA | Ligation of two fragments of 1.1kb fragment from pAC234 and 1.2kb fragment from pAC40 (G1^H381A^) with 5.3kb fragment from pSA25 (HindIII/XbaI) | 1.1kb fragment: ac_257F and ac_272R; 1.2kb fragment: ac_1F and ac_271R |
| G1-NTF-KDEL^ER^ | pAC270 | NTF of G1 with a C-terminal KDEL | HA | Ligation of 1.2kb fragment from pAC31 (G1) with 5.3kb of pSA25 (HindIII/XbaI) | 1.2kb fragment: ac_1F and ac_264R |
| dCirl-E^WT^-ECR-CalnTM^ER^ | pAC271 | ECR of *Drosophila* Cirl (isoform E) attached to the lumenal side of ER membrane by conjugation with calnexin transmembrane region (Uniprot: E1JH11) | HA | Ligation of two fragments of 0.4kb fragment from pAC233 and 2.2kb fragment from pJT2 (dCirl-E) with 5.3kb fragment from pSA25 (HindIII/XbaI) | 0.4kb fragment: ac_257F and ac_272R; 2.2kb fragment: ac_1F and ac_266R |
| dCirl-E^H668A^-ECR-CalnTM^ER^ | pAC273 | ECR of *Drosophila* Cirl (isoform E) containing GPS-mutated, cleavage-deficient (H>A) mutation, attached to the lumenal side of ER membrane by conjugation with calnexin transmembrane region | HA | Ligation of two fragments of 0.4kb fragment from pAC233 and 2.2kb fragment from pNH226 (dCirl-E^H668A^) with 5.3kb fragment from pSA25 (HindIII/XbaI) | 0.4kb fragment: ac_257F and ac_272R; 2.2kb fragment: ac_1F and ac_266R |
| dCirl-E^WT^-ECR-Fluo-CalnTM^ER^ | pAC272 | ECR of *Drosophila* Cirl (isoform E) attached to the lumenal side of ER membrane by conjugation with a mEmerald, followed by a calnexin transmembrane region | HA | Ligation of two fragments of 1.1kb fragment from pAC234 and 2.2kb fragment from pJT2 (dCirl-E) with 5.3kb fragment from pSA25 (HindIII/XbaI) | 1.1kb fragment: ac_257F and ac_272R; 2.2kb fragment: ac_1F and ac_266R |
| dCirl-E^H668A^-ECR-Fluo-CalnTM^ER^ | pAC274 | ECR of *Drosophila* Cirl (isoform E) containing GPS-mutated, cleavage-deficient (H>A) mutation, attached to the lumenal side of ER membrane by conjugation with a mEmerald, followed by a calnexin transmembrane region | HA | Ligation of two fragments of 1.1kb fragment from pAC234 and 2.2kb fragment from pNH226 (dCirl-E^H668A^) with 5.3kb fragment from pSA25 (HindIII/XbaI) | 1.1kb fragment: ac_257F and ac_272R; 2.2kb fragment: ac_1F and ac_266R |
| dCirl-E-NTF-KDEL^ER^ | pAC275 | NTF of *Drosophila* Cirl (isoform E) with a C-terminal KDEL | HA | Ligation of 2.2kb fragment from pJT2 (dCirl-E) with 5.3kb of pSA25 (HindIII/XbaI) | 2.2kb fragment: ac_1F and ac_267R |
| E2^WT^-ECR-2ht-CalnTM^ER^ | pAC280 | ECR of E2, followed by (EAAAK)_2_ α-helical turns and a calnexin transmembrane region | HA | Synthesised by Genscript | - |
| E2^WT^-ECR-4ht-CalnTM^ER^ | pAC281 | ECR of E2, followed by (EAAAK)_4_ α-helical turns and a calnexin transmembrane region | HA | Synthesised by Genscript | - |
| E2^WT^-ECR-6ht-CalnTM^ER^ | pAC282 | ECR of E2, followed by (EAAAK)_6_ α-helical turns and a calnexin transmembrane region | HA | Synthesised by Genscript | - |
| E2^WT^-ECR-8ht-CalnTM^ER^ | pAC283 | ECR of E2, followed by (EAAAK)_8_ α-helical turns and a calnexin transmembrane region | HA | Synthesised by Genscript | - |
| E2^WT^-ECR-10ht-CalnTM^ER^ | pAC284 | ECR of E2, followed by (EAAAK)_10_ α-helical turns and a calnexin transmembrane region | HA | Synthesised by Genscript | - |

**Supplementary Table 7. List of primers used in this study.**

| **Primer ID** | **Sequence (5’🡪3’)** |
| --- | --- |
| ac_1F | TAATACGACTCACTATAGGG |
| ac_2R | GGCACAGTCGAGGCTGATCAGCGGG |
| ac_7F | GCCGTTGCACCCGCCTGAGCAGC |
| ac_8R | GCTGCTCAGGCGGGTGCAACGGC |
| ac_9F | CCCGGATCCATGGGAGGCCGCGTCTTTCTCG |
| ac_10R | CACGGGATCTTCCTCCTGCACAT |
| ac_11F | ATGTGCAGGAGGAAGATCCCGTG |
| ac_21R | GCTCTAGCATTTAGGTGACAC |
| ac_54R | GTCGGGGACGTCGTAGGGGTAGGATCCGGGCCGGCCCTTG |
| ac_55F | TACCCCTACGACGTCCCCGAC |
| ac_66R | ATAGAATAGGGCCCTCTAGATCACAGGGATGTGACCAGGTG |
| ac_68R | ATAGAATAGGGCCCTCTAGATCAAACTTCTGTTTGAAAGTC |
| ac_89F | AAAGCCATCCAGTAGAACACCAGCACC |
| ac_90R | GGTGCTGGTGTTCTACTGGATGGCTTT |
| ac_142F | CCGGACACAAGGTGTAGCTGTGCTCCATC |
| ac_143R | GATGGAGCACAGCTACACCTTGTGTCCGG |
| ac_149R | GTGGTCAACTAGTACTCAAGCATC |
| ac_150R | GATGCTTGAGTACTAGTTGACCAC |
| ac_151F | GGAACACCTTCCTAGCGCTGCTGGC |
| ac_152R | GCCAGCAGCGCTAGGAAGGTGTTCC |
| ac_153F | CCTCAATAGTTAGGAAGTGTCCACC |
| ac_154R | GGTGGACACTTCCTAACTATTGAGG |
| ac_155F | CTGCCCGGGTCTAGATGGCCTACC |
| ac_156R | GGTAGGCCATCTAGACCCGGGCAG |
| ac_180F | CATGGCCCACTACGATGTGCAGCTGCTATCCGTGCCGTTGCTGC |
| ac_181R | TACCGTCGACTGCAGAATTC |
| ac_182R | GCAGCAACGGCACGGATAGCAGCTGCACATCGTAGTGGGCCATG |
| ac_183F | CATGGCCCACTACGATGTGCAGGACCGGTCGATGGTGAGCAAG |
| ac_184R | CTTGCTCACCATCGACCGGTCCTGCACATCGTAGTGGGCCATG |
| ac_185R | TACCGTCGACTGCAGAATTCTTACTTGTACAGCTCGTCCATGC |
| ac_187R | ATAGAATAGGGCCCTCTAGATCACAGGTGGGTGCAACGGCAGATG |
| ac_188R | ATAGAATAGGGCCCTCTAGATCACAGCTCGTCCTTCAGGTGGGTGCAACGGCAGATG |
| ac_197F | CAGATTATGCTGGGGCCCTCTACCCCTACGACGTCCCCGAC |
| ac_198R | ATGAGTTTTTGTTCGTCGACCTGCACATCGTAGTGGGCCATG |
| ac_249F | GACCGGTCGTGGCTGTGGGTAGTCTATAT |
| ac_257F | CTGCTATCCGTGCCGTTGCTGC |
| ac_258R | GCAGCAACGGCACGGATAGCAGCCTGGTGATCAGGGTCAGCTTCC |
| ac_259R | GCAGCAACGGCACGGATAGCAGGACTGACAGCAACAGCTCATTAA |
| ac_260R | GCAGCAACGGCACGGATAGCAGCACGTCCAGCAGCAGATCGTGC |
| ac_261R | ATAGAATAGGGCCCTCTAGATCACAGCTCGTCCTTCAGGTGGCTGCATTGGCAGG |
| ac_262R | ATAGAATAGGGCCCTCTAGATCACAGCTCGTCCTTGAGGTGGCTGCAGGCACATG |
| ac_263R | ATAGAATAGGGCCCTCTAGATCACAGCTCGTCCTTCAGGTGATTACAGCTGCATG |
| ac_264R | ATAGAATAGGGCCCTCTAGATCACAGCTCGTCCTTCAAGTGGTTGCAGAAGCAGG |
| ac_265R | ATAGAATAGGGCCCTCTAGATCACAGCTCGTCCTTGAGGTGAGTGCAGCGGCAGAC |
| ac_266R | GCAGCAACGGCACGGATAGCAGGCGCATGTTTCCATCGAACATGG |
| ac_267R | ATAGAATAGGGCCCTCTAGATCACAGCTCGTCCTTCAGGTGGTTGCAACTGCAGAC |
| ac_270R | GCAGCAACGGCACGGATAGCAGGCTGATAGACGACAGCGCCACC |
| ac_271R | GCAGCAACGGCACGGATAGCAGGAGGCTCAGGTAGTGCTTGTGC |
| ac_272R | TATAGAATAGGGCCCTCTAG |
| ac_336F | CCCGTGCTGACTGTCATCACCATCACCTGGGTGGGGATTCTGC |
| ac_337R | GGTGATGACAGTCAGCACGGG |
| ac_338F | CCCGTGCTGACTGTCATCACCTATGTGGGCTGCTCCCTCTCCG |
| ac_339F | CCCGTGCTGACTGTCATCACCCTCTCCTACGTGGGCTGTGTCG |
| ac_362F | CCCGTGCTGACTGTCATCACCGACGTCACGCAGCAAAGGGACG |
| ac_363R | ATAGAATAGGGCCCTCTAGATTACAGCAGTGAGTCATTTGTAC |
| ac_364F | CCCGTGCTGACTGTCATCACCTACAACTACTATGCCACACTGC |
| ac_365R | ATAGAATAGGGCCCTCTAGATCAGCAGTGGAGGATCTTCAGG |
| ac_366F | CCCGTGCTGACTGTCATCACCTTCCCACTGCTCTACACTGTCC |
| ac_367R | ATAGAATAGGGCCCTCTAGATTACTTATCGTCATCGTCC |
| ac_368F | CCCGTGCTGACTGTCATCACCCTAATAGTAGGCAGTGGTC |
| ib_39F | GTTGCACCCACCTGGCCAGCTTTGCCGTC |
| ib_40R | GACGGCAAAGCTGGCCAGGTGGGTGCAAC |
| ib_45F | CTGCCGTTGCACCGCCCTGAGCAGCTTTG |
| ib_46R | CAAAGCTGCTCAGGGCGGTGCAACGGCAG |
