# Supplementary material for: Self-cleavage of the GAIN domain of adhesion G protein-coupled receptors requires multiple domain-extrinsic factors
Source: Nat Commun. 2025 Oct 1;16:8736. doi: 10.1038/s41467-025-64589-3 (PMC12488864; doi:10.1038/s41467-025-64589-3)
Supplement: Supplementary file 2 — Description of Additional Supplementary Files [file 41467_2025_64589_MOESM2_ESM.pdf]

Description of Additional Supplementary Files

File name: Supplementary Movie 1

Description: Timelapse tracking of SBP-eGFP-E2^{WT}-7TM transport in RUSH assay from ER to plasma membrane. Receptor trafficking was induced by addition of biotin (40 μM). Fluorescence was detected every 5 minutes. Location of receptor was monitored by the eGFP signal, indicated in purple. ER was visualised by CellLight BacMam 2.0 and highlighted in green. Nuclei of the cells are stained by Hoechst 33342 and shown in cyan. Movies were digitally adjusted in brightness and contrast to enhance the visibility of the signals. Scale bar, 10 μm.

File name: Supplementary Movie 2

Description: Timelapse tracking of SBP-eGFP-E2^{WT}-7TM transport in RUSH assay from Golgi to plasma membrane. Receptor trafficking was induced by addition of biotin (40 μM). Fluorescence was detected every 5 minutes. Location of receptor was monitored by the eGFP signal, indicated in purple. Golgi was visualised by CellLight BacMam 2.0 and highlighted in green. Nuclei of the cells are stained by Hoechst 33342 and shown in cyan. Movies were digitally adjusted in brightness and contrast to enhance the visibility of the signals. The Z-plane was adjusted along the movie to maintain the receptor population continously in the focal plane. Scale bar, 10 μm.
