# Supplementary material for: Self-cleavage of the GAIN domain of adhesion G protein-coupled receptors requires multiple domain-extrinsic factors
Source: Nat Commun. 2025 Oct 1;16:8736. doi: 10.1038/s41467-025-64589-3 (PMC12488864; doi:10.1038/s41467-025-64589-3)
Supplement: Supplementary file 5 — Reporting Summary [file 41467_2025_64589_MOESM5_ESM.pdf]

nature portfolio

Corresponding author(s): Tobias Langenhan

Last updated by author(s): Sep 1, 2025

Reporting Summary

Nature Portfolio wishes to improve the reproducibility of the work that we publish. This form provides structure for consistency and transparency in reporting. For further information on Nature Portfolio policies, see our Editorial Policies and the Editorial Policy Checklist.

Statistics

For all statistical analyses, confirm that the following items are present in the figure legend, table legend, main text, or Methods section.

n/a	Confirmed	
☐	☒	The exact sample size (*n*) for each experimental group/condition, given as a discrete number and unit of measurement
☐	☒	A statement on whether measurements were taken from distinct samples or whether the same sample was measured repeatedly
☐	☒	The statistical test(s) used AND whether they are one- or two-sided<br>*Only common tests should be described solely by name; describe more complex techniques in the Methods section.*
☒	☐	A description of all covariates tested
☐	☒	A description of any assumptions or corrections, such as tests of normality and adjustment for multiple comparisons
☐	☒	A full description of the statistical parameters including central tendency (e.g. means) or other basic estimates (e.g. regression coefficient) AND variation (e.g. standard deviation) or associated estimates of uncertainty (e.g. confidence intervals)
☐	☒	For null hypothesis testing, the test statistic (e.g. *F*, *t*, *r*) with confidence intervals, effect sizes, degrees of freedom and *P* value noted<br>*Give P values as exact values whenever suitable.*
☒	☐	For Bayesian analysis, information on the choice of priors and Markov chain Monte Carlo settings
☒	☐	For hierarchical and complex designs, identification of the appropriate level for tests and full reporting of outcomes
☒	☐	Estimates of effect sizes (e.g. Cohen's *d*, Pearson's *r*), indicating how they were calculated

Our web collection on statistics for biologists contains articles on many of the points above.

Software and code

Policy information about availability of computer code

Data collection	*Provide a description of all commercial, open source and custom code used to collect the data in this study, specifying the version used OR state that no software was used.*
Data analysis	The following software packages were used in this study: ImageJ2/Fiji v2.9.0/1.53t; Zeiss ZEN; Leica LAS X suite; Prism 8.4.3

For manuscripts utilizing custom algorithms or software that are central to the research but not yet described in published literature, software must be made available to editors and reviewers. We strongly encourage code deposition in a community repository (e.g. GitHub). See the Nature Portfolio guidelines for submitting code & software for further information.

Data

Policy information about availability of data

All manuscripts must include a data availability statement. This statement should provide the following information, where applicable:

- Accession codes, unique identifiers, or web links for publicly available datasets
- A description of any restrictions on data availability
- For clinical datasets or third party data, please ensure that the statement adheres to our policy

The mass spectrometry proteomics data have been deposited to the ProteomeXchange Consortium via the PRIDE partner repository with the dataset identifier PXD056561. The uncropped scans for all Western blots shown in this study are available in figshare at https://figshare.com/s/b8448b96473d8b7de21a. The processed data generated in this are provided in the Source data file.

Human research participants

Policy information about studies involving human research participants and Sex and Gender in Research.

Reporting on sex and gender	N.A.
Population characteristics	N.A.
Recruitment	N.A.
Ethics oversight	N.A.

Note that full information on the approval of the study protocol must also be provided in the manuscript.

Field-specific reporting

Please select the one below that is the best fit for your research. If you are not sure, read the appropriate sections before making your selection.

☒ Life sciences ☐ Behavioural & social sciences ☐ Ecological, evolutionary & environmental sciences

For a reference copy of the document with all sections, see nature.com/documents/nr-reporting-summary-flat.pdf

Life sciences study design

All studies must disclose on these points even when the disclosure is negative.

Sample size	No sample size calculation was performed. For all assay replicates were performed as indicated in the related figure caption.
Data exclusions	No data were systematically excluded.
Replication	Each experiment was reliably reproduced on separate occasions, number of replicates and sample sizes of displayed results are indicated in the figure legends.
Randomization	Allocation of samples (cells) for data collection and analyses was random. Covariates were not relevant in this study as experimental and control experiments were performed in parallel, and cells were maintained under identical rearing/culture conditions.
Blinding	Blinding for ELISA, Western blotting and luciferase analyses was not necessary due to quantitative nature of the experiment. Statistical analyses for MS were blinded, with the person performing the experiment not knowing the rationale of the experimental design.

Reporting for specific materials, systems and methods

We require information from authors about some types of materials, experimental systems and methods used in many studies. Here, indicate whether each material, system or method listed is relevant to your study. If you are not sure if a list item applies to your research, read the appropriate section before selecting a response.

Materials & experimental systems

n/a	Involved in the study	
☐	☒	Antibodies
☐	☒	Eukaryotic cell lines
☒	☐	Palaeontology and archaeology
☒	☐	Animals and other organisms
☒	☐	Clinical data
☒	☐	Dual use research of concern

Methods

n/a	Involved in the study	
☒	☐	ChIP-seq
☒	☐	Flow cytometry
☒	☐	MRI-based neuroimaging

Antibodies

Antibodies used	Primary antibodies<br>rat α-HA-Peroxidase (Roche, clone 3F10, #12013819001)<br>mouse α-HA-AlexaFluor-647 (Invitrogen, RRID: AB_2610626)<br>mouse α-tubulinβ (DSHB e7, RRID: AB_528499)<br>rabbit α-HA (Cell Signaling Technology, #C29F4; RRID:AB_1549585)

	rabbit α-RFP (ChromoTek, RRID:AB_2631395)<br>rabbit α-myc (invitrogen, RRID: AB-2849731)<br><br>Secondary antibodies<br>IRDye 680RD goat-α-rabbit (RRID:AB_2721181)<br>IRDye 800CW goat-α-mouse (RRID:AB_2687825)
Validation	Empty-vector-transfected cells were always included in all experiments to ensure the specificities of the primary antibodies used. We also used α-tubulinβ for the detection of tubulinβ as a loading control for Western blotting.

Eukaryotic cell lines

Policy information about cell lines and Sex and Gender in Research

Cell line source(s)	HEK293T, German Collection of Microorganisms and Cell Culture (Braunschweig, Germany), #ACC635
Authentication	Cell lines were maintained and authenticated by the supplier, no additional authentication was performed by the authors of this study.
Mycoplasma contamination	Cell lines were regularly tested for mycoplasma contamination by the authors and tested negative.
Commonly misidentified lines<br>(See ICLAC register)	No commonly misidentified cell line was used in this study.
